# Stress Concepts and Applications in Various Matrices with a Focus on Hair Cortisol and Analytical Methods

**DOI:** 10.3390/ani12223096

**Published:** 2022-11-10

**Authors:** Jalil Ghassemi Nejad, Morteza Hosseini Ghaffari, Mohammad Ataallahi, Jang-Hoon Jo, Hong-Gu Lee

**Affiliations:** 1 Department of Animal Science, Konkuk University, Seoul 05029, Korea; 2 Institute of Animal Science, University of Bonn, 53115 Bonn, Germany; 3 College of Animal Life Sciences, Kangwon National University, Chuncheon 24341, Korea

**Keywords:** stress, hormones, biomarkers of stress, matrix, domestic animals

## Abstract

**Simple Summary:**

Stress in domestic animals can lead to serious consequences. We reviewed biological specimens (fluid and non-fluid types), that are capable of evaluating either cortisol or corticosterone steroid levels as major biomarkers of stress (acute or chronic) in different animal species. Proper methods to evaluate the chronic stress of animals through hormonal analysis can save time and cost of experiments in clinical laboratories due to different research groups applying different techniques for preparation, extraction, and analysis of the biological specimens that may result in fluctuating and inaccurate values. In addition, we reviewed the immunoassays such as singleplex immunoassays (ELISA) and multiplex immunoassays as the most common method used for detecting multi-biomarkers of stress. In this review, we aimed to: (1) explain the classification of stress, (2) discuss the matrices that can be used as biomarkers of stress, their comparison, and limitations, and present the most important reliable matrix, (3) compare the analytical methods for measuring stress hormones after sample preparation to determine the result. In this review, we have discussed the method of sample collection, sex and age effects, the body regions to be selected, and the method of analysis of cortisol and corticosterone in different body indices that may be used from time to time. We also compared the advantages and disadvantages of each matrix and technique for analyzing stress hormones.

**Abstract:**

When studying stress in animals, it is important to understand the types of stress and their classification, and how to assess the stress levels in different animal species using different matrices accurately and precisely. The classification of stress types helps to distinguish between good stress (eustress) and bad stress (distress). Hence, first, it is crucial to assess the animal’s level of stress in a non-intrusive manner and second to identify the type of stress that is best suited to its environment. Third, it is also important to analyze the obtained samples using a suitable method to increase the validity of stress hormone measurements. Therefore, in this review, we aim to: (1) explain the classification of stress, (2) discuss the wide range of body matrices (e.g., saliva, milk, hair, urine, feces, sweat, fins, etc.) that can be used as samples to evaluate stress levels, as well as their comparisons and limitations, and present the reliable matrices for measuring stress hormones with special emphasis on hair, (3) compare the analytical methods for measuring stress hormones after sample preparation. Despite some literature that does not include hair as a reliable matrix for evaluating stress levels, hair is one of the matrices for measuring long-term stress hormone accumulations. This review discusses some factors that influence the level of stress hormones in the hair. By understanding these issues, the scientific community will not only be able to improve the understanding of stress and biomarker evaluation but also suggest how to deal with the consequences of stress in future research.

## 1. Introduction

### 1.1. Stress Definitions

In general, the condition of a series of physiological and behavioral changes triggered by increased activation of the hypothalamic-pituitary-adrenal (HPA) axis leading to the release of stress hormones in the blood is called stress [1]. There is an attempt to maintain homeostasis of the body regulated by the sympatho-adrenomedullary system (SAM) while animals are exposed to various stimuli or stressors [1]. Stress can be measured through the aforementioned SAM and the HPA axis, as they are the two main components of the stress response and the increase of stress hormones in the body (blood). These two pathways are critical for energy metabolism [1,2]. Animals can be affected by stress in different ways, but there are mainly two types: stress that has a positive effect and that can be positive, namely eustress [3,4] and stress that has a negative effect on the body (e.g., poor performance, animal welfare problems, health issues, etc.); distress [3,5]. Thus, not all types of stress can be harmful or even negative [3]. In response to actual stressful stimuli (controlled eustress; uncontrolled distress), the body provides additional energy to cope with the stressful state by activating the sympathetic nervous system (SNS), resulting in the release of stress hormones and chemicals such as epinephrine and cortisol into the bloodstream [6]. 

In this review, we discuss factors that influence the concentration of stress hormones, with special attention to hair as a reliable matrix for measuring long-term stress hormones. To better manage the consequences of stress in animals, we should better understand the level of stress, the assessment of stress or stressors, the type of stress, the type of stress stimuli (stressors), the factors affected by stress, and the physiological status of the animals (i.e., sex, age, body weight, etc.).

### 1.2. Stress Classifications

Acute and chronic stress depend on different sources of stressors [7,8,9], which have been classified as sequential, episodic, chronic, intermittent, persistent, or anticipatory [3,10]. Depending on the condition concerning the duration of the effects, stress is referred to as either short-term or long-term stress [3,9]. When the central nervous system (CNS) perceives a threat, a series of general biological defense responses become active in the animal to respond to the threat. An acute stress response usually occurs after the animal perceives a brief threat, either physical, emotional, or psychological. Then, the animal’s physiological balance recovers quickly, leading to a full adaptation [11]. During an acute stress response, the HPA axis is activated, and many hormones are released within seconds or minutes, leading to physiological and metabolic effects such as increased heart rate, respiratory rate, blood pressure, intestinal flow rate, energy mobilization, stimulating immune function, decreasing appetite, decreasing some plasma minerals (potassium, magnesium), and slowing the digestive flow in the rumen and stomach [3].

In contrast to acute stress, chronic stress is an event of ongoing physiological arousal [9,12] and occurs when the body experiences multiple stressors or repetitions of acute stress responses such that the autonomic nervous system is unable to activate normal physiological and behavioral adaptations [13,14]. In general, the long-term overstimulation of coping responses leads to direct effects such as increased body heat, low energy, and anxiety or indirect effects such as changes at the functional level of the endocrine system, immune system, and metabolic system. These effects lead to prepathological or pathological consequences that affect health and welfare [13,15]. While acute stress results in a rapid and fairly complete recovery of physiological balance (adaptation), chronic stress prevents animals from fully recovering from prolonged stressful conditions (maladaptation) [3]. Response due to acute stress, which occurs when an animal feels threatened, causes the body to release various stress hormones such as cortisol, corticosterone, and adrenaline (also known as epinephrine) into the bloodstream. In most animals, the release of these hormones over time can lead to serious health problems. Frequent, intense, or chronic stress is toxic to the body and brain and has been linked to several physiological disorders [16], impaired productivity, behavior, and animal well-being [17]. With that said, we attempted to provide information in the following chapter about the various biomarkers of stress and how the stress hormone can be measured in different biomatrices that can be used in different animals based on the ease of use and applicability of each sample’s nature. 

## 2. Biomarkers of Stress in Different Animal Species

### 2.1. Biomarkers of Stress

Among biomarkers, it is crucial to always identify the reliable ones depending on the applicability of the sample, as the markers must be highly correlated with the specific pathophysiological aspects of the particular stress [9,18]. In general, biomarkers for stress may include proteins, enzymes, hormones, chemicals, metabolites, genes, or byproducts [9]. Under stressful conditions, two major endocrine systems are activated [9,13], resulting in the release of various hormones, including epinephrine, norepinephrine, and cortisol [18,19,20]. Cortisol and corticosterone are the primary glucocorticoids and have been used as classic biomarkers of stress in animals [7,8,9,21]. Although cortisol and corticosterone are both detectable in many animal species, cortisol is the primary endogenous adrenal steroid in most mammals, including humans, many larger mammals, vertebrates, and fish, while corticosterone is the primary adrenal corticosteroid in few rodents, and birds to understand biological systems [9]. In research experiments or medical diagnostics, the majority of cortisol assays have been performed on biomatrices such as serum, saliva, urine, milk, and other biological fluids. However, most of these liquid biomatrices are suitable for measuring cortisol concentration at a single time point and represent the acute stress state in the physiological diurnal fluctuations. 

Over the past decade, the use of hair as a biomarker of stress has been well-documented and established [9]. Taken together, we now focus on the availability and usability of hair cortisol as a measure of stress in domestic animals and mention that wool and fleece also fall into this category. Several non-liquid biomatrices including hair [7,8], feather [9,22,23,24], fin [21], wool [8], turtle claws [25], dog nails [26], cat nails [27], feces [28,29,30,31], nails [32], tooth [33] are applied for measuring overall long-term systemic glucocorticoids (cortisol, corticosterone) exposure. These biomatrices can be used to monitor chronic stress levels, and noninvasive, stress-free sampling is particularly beneficial to animal well-being. For example, in healthy animals, blood cortisol levels can fluctuate (blood cortisol levels peak in the early morning and gradually decline thereafter) [34], and in addition, factors such as low or hot temperatures, humidity, and wind can affect blood cortisol levels. In addition, cortisol or corticosterone levels in biomatrices such as serum and saliva reflect HPA axis function shortly after its activation, and in biomatrices such as urine and feces reflect HPA activity ranging from a few hours to a few days before measurement. Feces can be used for monitoring both acute and chronic stress. That is not a matter of the material, but the frequency of sample collection. Capturing the acute response requires more frequent samples (in order not to miss the peak excretion of fecal cortisol metabolites), while chronic stress (or baseline HPA activity) can be assessed by a few samples (especially because levels are smoothened in the feces) [9,28,30]. However, cortisol or corticosterone levels in biomatrices such as hair, feathers, fins, scales, nails, and teeth reflect HPA activity over longer periods (weeks or even months), making non-liquid biomatrices useful for estimating chronic stress in animals and humans [9]. However, the use of alternative biomatrices in different animal species depends on the objectives of the study [21,22,32]. Therefore, all established alternative matrices as well as the exploration of new alternative stress indicators should be considered when studying stress in animals.

There is a potential relationship between cortisol levels in hair and those in saliva, urine, and feces, but each of these has its limitations [9,35]. Furthermore, correlations between different samples of materials have not proven useful, especially those reflecting different time windows. Nonetheless, the trend (of increased or decreased) stress hormones in various matrices provide insights into interpreting the results of studies which they can be studied by comparing the level of hormones in different matrices (e.g., saliva vs. blood, hair, etc.) [9]. Given the variety of the research analyzed, meta-analyses of correlation coefficients showed significant variability between studies [36]. There are a number of factors that tend to bias the result of blood cortisol and its metabolites rather than the treatment effect, which can also be used in the same manner to alter saliva values. It should be noted that while the use of urine and feces for hormone measurement is promising and associated with hormone production over an extended period, collecting urine and feces samples from each individual and the difficulty of storing these samples pose some difficulty [9]. It seems that the use of hair, wool, and feathers from pets to better identify hormonal changes over time is a better approach to overcoming the above difficulties [9]. Earwax has been recently presented as a promising matrix that can be reliably used to measure stress by extracting cortisol. Earwax has the same advantages of hormone measurement and sampling in hair/wool/feathers over traditional biological fluids (blood, plasma, serum, and saliva) and eliminates ethical concerns because sampling is non-invasive [37]. The recent indicator of cortisol in earwax as a non-stressful sampling method in both humans [38] and animals [no published data] is also considered to provide cumulative retrospective measurements of stress hormones (up to a few weeks) and sampling is simple and not painful. Earwax is thought to represent an accumulation of cortisol output over weeks or months [9]. In addition, the collection and storage of earwax and hair are simple, which may facilitate their use in chronic stress research. In addition, the scales, fins, and jawbones of aquatic animals appear to be promising matrices to show stress levels by measuring cortisol levels [9,21]. However, further studies are needed to validate these matrices as a reliable indicator of stress. A comparison of the hair matrix with other biomatrices that have been used to study chronic stress in animals is shown in Table 1 [37,39]. In addition, a classical biomarker should have the following characteristics: (1) ease of collection and processing of the appropriate biological sample; (2) stability and durability of the marker throughout the storage and evaluation period; (3) availability of assays with sufficient specificity and sensitivity for the marker in question. Additionally, the identification of non-invasive methods for biomarker assessment has the potential to provide accurate data concerning induced stress stimuli to ensure a standard measurement in response to stress.

### 2.2. Evaluation of Stress in Domestic Animals Including Ruminants, Birds, and Aquatic Organisms

Using blood cortisol and corticosterone (serum and plasma) as a biomarker of stress can be confounded and problematic due to its possible affection by various factors [40,41,42,43,44,45,46], including circadian rhythms [43]; sampling [47,48]; restraint [42,43,49]; stage of lactation [50]; milking [42,49,51], degree of habituation [52,53], other hormones (e.g., vasopressin can potentiate ACTH secretion [51]; infections as well as endotoxins [3,51]. The phenomenon could also be extended to salivary cortisol. Thus, in the case of the use of catheters, the restraining issue can be solved, while the use of other biomarkers can contribute to a better interpretation of the results. The use of saliva as a biological sample has advantages including being non-invasive in some animal species [54], particularly in pigs where blood collection is stressful and painful for both the animal and the staff in charge of the sampling [54,55]. Cortisol exists in two fractions in blood: protein-bound cortisol and free cortisol, whereas free cortisol is the active fraction only in saliva [54,55]. In aquatic animals, recent studies focused on the measurement of cortisol in golden fish [56] and sturgeon [21] and indicated the usability of fins and scales as matrices to extract and show cortisol levels. Recently, Ghassemi Nejad et al. [21] took a step forward and successfully extracted cortisol from the jawbones of sturgeons. They also identified the type of washing solvent and its effect on cortisol detection and found that there were no differences in cortisol levels in fins of three sturgeon species or cortisol levels in the fins and jawbones between washing solvents. Overall, they suggested that the sturgeon jawbone matrix is a promising alternative stress indicator to non-liquid matrices in dead fish. 

## 3. Recent Advances in Stress Evaluation Indexes

### 3.1. Measuring Hair Cortisol

In reviewing many matrices that can be used as biomatrices of stress, hair is one of the most reliable matrices for assessing stress levels by measuring the hormones cortisol and corticosterone. Measuring glucocorticoids (GCs) deposited in hair is a popular biomarker-based stress assessment method. On the other hand, while hair can be easily and painlessly sampled, it has been argued that it has superior qualities to other methods for analyzing GCs when it comes to gauging chronic stress [57,58,59]. The enzyme 11β-hydroxysteroid dehydrogenase (11-HSD) is a bifunctional enzyme which is determined by the NAD(H)/NAD ratio [60]. Cortisol is converted into cortisone in hair by 11-HSD isoenzyme type 2, resulting in higher cortisol concentrations in samples, as shown in human and sheep studies [61,62,63]. The activity of 11-HSD type 2 in hair, measured as the cortisol to cortisone ratio, has been used as a stress biomarker in humans [64,65]. López-Arjona et al. [66] reported that hair cortisol levels and the cortisol/cortisol ratio (an estimate of 11-HSD isoenzyme type 2 activity) increased more than cortisol after farrowing in pigs, with the magnitude of these changes being greater during periods of higher atmospheric temperature. Furthermore, animal hairs are different from human ones, because they do not grow continuously. Hair cortisol levels track blood levels over time, so hair can collect information on how cortisol levels have changed over time, making it a suitable biomatrix for monitoring the well-being and health of animals exposed to environmental and physiological stressors. Therefore, at this point, this report focuses more on the extraction and measurement of hormones from hair. More recently, researchers have reported that analyzing cortisol levels in animal species exposed to prolonged periods of stress (chronic stress) is a difficult task because no biomatrices are representing long-term systemic cortisol levels that can be measured unless a solid matrix such as hair is used, in which the hormone gradually accumulates [9]. Therefore, hair samples have been used to analyze long-term cortisol levels and provide an accurate index of average systemic cortisol [67,68]. Taken together, analysis of hair to detect cortisol has many potential applications, including monitoring chronic stress in humans and other animals to detect suboptimal environments and management, it can be used to predict disease risk or track endocrine disorders (e.g., Cushing’s disease), monitor psychological disorders (e.g., depression and anxiety), and genetic selection or management of animals [2,18]. 

### 3.2. Hair Biomatrix, Structure, and Mechanism for Incorporation of Hormone

**Figure 1:**
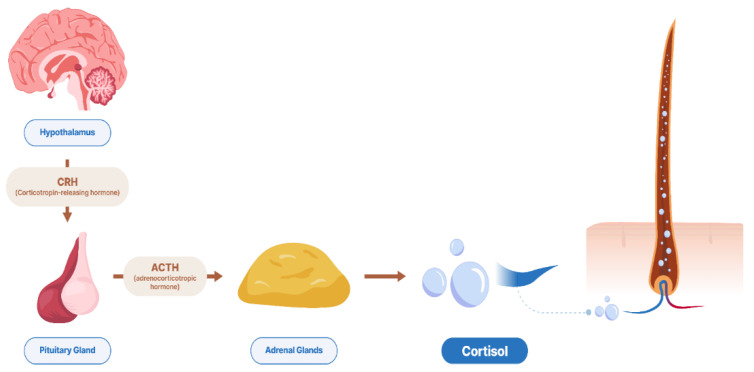
Infographic of cortisol incorporation to hair matrix from blood (Source: CO-ANI).

The infographic of cortisol incorporation into the hair via blood is shown in Figure 1. Chronic stress has negative effects on animal welfare and leads to an increase in cortisol levels, which can be measured in hair. Cortisol in the hair matrix has been detected and provides valuable information about the long-term activity of the HPA axis in chronic stress situations [35,59,69,70], and no other biomatrix seems to have the same potential feature. According to Arck et al. [71], cortisol in hair could originate from the blood, skin, or follicle and regulate follicular function, which is a possible explanation for using cortisol in hair as a biomatrix for stress exposure. Therefore, hair has become an important specimen in research experiments and medical diagnoses.

Hair is a type of keratinous filament that grows out of the epidermis in mammals [72]. The structure of the hair matrix consists of different parts, but in general, it is formed by keratinized cells and has two main parts: the hair shaft and the hair follicle [72,73]. The hair follicle is a hair strand that originates in an epidermal penetration of the dermis. The part of the hair that is not anchored in the follicle but is largely exposed on the skin surface is called the hair shaft, and the hair root refers to the remainder of the hair that is anchored in the follicle and lies below the skin surface. The part of the hair root that ends deep in the dermis in the area of the hair bulb is called the hair matrix, which contains a layer of mitotically active basal cells. The hair papilla is the part of the connective tissue that contains blood capillaries and nerve endings from the dermis. The hair papilla is surrounded by the hair bulb.

According to Boumba et al. [72], human hair is composed of proteins (65–95%), water (15–35%), lipids (1–9%), and minerals (0.25–0.95%). Wennig [74], studied the growth of head hair while analyzing drug exposure in human hair and reported that hair grew an average of 1 cm per month [75,76]. The growth rate of hair should be considered before planning the experiment and haircut intervals. Human scalp hair grows on average at about 1 cm per month on average, although there is considerable individual variation [75,76]. Heimbürge et al. [75,76], reported the range of hair growth from 5.3 to 12.0 mm per month in pigs and the range of hair growth from 3.5 to 17.0 mm per month in cattle during the study of factors affecting the level of cortisol in the hair of cattle and pigs. In addition, Burnett et al. [77], reported that the rate of hair growth was faster at the tail tip than at the hip and shoulder areas of the cattle body. In addition, other studies examined the rate of hair growth from an average of 7 mm to 27 mm per month in different primates [75,77,78,79]. The explanation for these differences in hair growth rates may be divergent hair types, different hair lengths, or differences in skin-blood flow in the body region of the animals. Therefore, when analyzing hair cortisol levels, it is important to know the rate of hair growth in different species and also to assess the causes of variation, especially when hair cortisol is used as a retrospective biomarker of stress [76]. In conclusion, determining the rate of hair growth in different body regions of animal species is essential for tracking cortisol levels as a classic biomarker of chronic stress.

The hair technique is becoming a universal and promising tool for monitoring cortisol levels as an alternative method compared to traditional techniques (e.g., blood, saliva, urine). Since hair grows an average of 1 cm per month [74,75,76], it is suitable for measuring cortisol over a month. There are several explanations for the presence of cortisol in hair. Russel et al. [39] mentioned that cortisol could enter the hair mainly at the level of the medulla of the hair shaft by passive diffusion from the blood tissue. The hypothesis behind this idea is that the cortisol in hair reflects the integrated unbound cortisol fraction rather than the total amount of cortisol in serum. Another possibility is that cortisol from sebaceous and eccrine secretions coats the outer cuticle [39,80]. In addition, cortisol could be taken up into the hair by external contamination after the hair shaft has formed. Overall, there are four general models for the uptake of cortisol into hair [80]: I) by active or passive diffusion from the blood into the growing cells in the hair follicle. II) by diffusion from body secretions (e.g., sweat, sebum) during hair shaft formation. III) by uptake from the deep skin structure during the formation of the hair shaft and IV) by external environmental sources after the formation of the hair shaft.

Numerous studies have shown that there are many factors that influence the amount of cortisol in the hair. Knowledge of these factors makes it possible to correctly interpret the result. According to Baier et al. [81], these factors include hair color [77,82], hair collection technique, body region collection [57,77], age of the animal [83], pregnancy [84], season [69,85], and weather variations [85]. In addition, there are physical activities such as long-distance transport [86] and, in some cases, castration [87], excitability [88], and the use of fistula in cattle [81].

Hair cortisol provides information on the abnormalities of animals suffering from the disease over several months. Compared to conventional matrices such as blood or urine, sampling the hair matrix is a noninvasive method, it does not require experience or expertise to perform, and it is not limited to living animals it can be sampled from non-living animals. Furthermore, in certain circumstances, hair may be the only sample available for examination, such as in studies of skeletal remains or traumatic injuries [89]. This aspect makes the hair matrix much more suitable than other biomatrices for monitoring animal health. The strengths and considerations for measuring cortisol in hair samples are summarized in Table 2 [9,90].

### 3.3. Considerations for Hair Samples Collection

As previously stated in this review, regardless of the type (acute or chronic) or source of stress (physiological, environmental, etc.), all types play a role in increasing the secretion of GCs such as cortisol and corticosterone in the bloodstream, resulting in stress elevation. The GCs are commonly referred to as stress hormones, and they play a role in a variety of physiological processes [28]. Even though GC levels do not equate to stress levels, because GCs are important mediators of the physiological stress response, their determination is frequently used as a stress marker [28,75]. For stress assessment, it is important that the process of sampling must not affect the stress biomarker [75,76]. Some criteria need to be considered before beginning an experiment regarding the collection of hair samples from the animals’ bodies. It has been found that age, gestation, hair color, body region, methods of sample collection, sex, and season can affect hair cortisol levels [75,76]. 

#### 3.3.1. Areas of the Collection

The body region from which hair samples are collected may have an impact on cortisol level estimation. For example, some studies found that hair sampled from the tailhead had higher cortisol levels than hair sampled from the animal’s forehead and shoulder [57,77], while others found no significant difference in cortisol levels between hair sampled from the animal’s forehead, shoulder, and hip [91]. Several studies in domestic animals, including cattle [57,77], horses [92], pigs [93], and others found differences in hair cortisol levels based on body region. The proportion of hair follicles, as well as contamination by feces, grooming, and differences in hair growth rates and skin blood flow, could all be potential influencing factors for region-specific differences [57,93,94,95]. The general recommendation for hair sampling, on the other hand, is to collect the samples from a clean site on the body, and the same site should always be considered for the collection of newly grown hair [8,9]. Regarding the length of the sample, a length of 3 cm is recommended, where each cm corresponds to 1 month of hair growth in humans. However, in different animals, the rate of hair growth is different and depends mainly on the nutritional status and season. Our observations on the farm have shown that hair growth is slower in winter (cold season) than in summer (hot season) in cattle. 

#### 3.3.2. Color of the Samples and Coats

Cortisol levels are thought to be affected by coat color [7,96,97], while hair color showed differences in cortisol levels in dogs [98], but not in cattle [7]. In the previous study, we found that white coat colors retained less cortisol than black coat colors, and no significant differences were observed in hair cortisol levels between black and white hair, which stored less cortisol, in both Holstein heifers and dairy cows under heat stress [7]. Most recently, we found higher hair cortisol in white coat color cows during cold the winter than in black color cows [99]. The protein that determines coat color utilizes the melanocortin signaling pathway, which regulates the amount of cortisol production [99,100]. The signaling pathway determines skin and hair color and is related to adaptation to stress. Mammals share developmental, physiological, and biochemical similarities in glucocorticoid and pigment production [7,101,102], which involves melanocyte-stimulating hormone and melanocortin receptors that regulate hair coat and color [99]. The melanin-based coloration process is controlled by the transmembrane G-protein-coupled melanocortin-1 receptor (MC1R) and its melanocortin agonist, melanin-stimulating hormone (α-MSH), and antagonist, agouti protein [99,100]. Circulating α-MSH stimulates MC1R in melanocytes, which in turn leads to an intracellular increase in cyclic adenosine monophosphate levels. Several domesticated breeds exhibit this color pattern, including dogs, pigs, horses, and cows [103,104]. To date, the conflicting findings on cortisol levels in dark and light hair have not been fully elucidated but could be related to physical space within the hair shaft, increased blood flow to skin covered by black hair [99], interactions with melanin, or greater washout in darker hair due to UV radiation [76,77,80,105,106]. Overall, the studies on the influence of hair color on hair cortisol level show conflicting results, and the underlying mechanisms of cortisol uptake in different-colored hair need further investigation.

#### 3.3.3. Method of Sample Collection, Preservation, and Preparation for Hormone Extraction

There are several methods for sample collection. The standard method is to cut the hair from the cleanest area (to avoid contamination by urine, feces, dust, etc.) with a razor or clippers as close to the skin as possible. There are reports of using scissors [8], but to obtain a homogeneous cut, this is not advisable [107]. The same site should be marked to cut the newly grown hair and measure the hormones in the new cut. The old cut can be used as a baseline, but not to see the effects of the treatment. Immediately after collection, and to avoid environmental contamination, each hair sample should be placed in aluminum foil (unaffected by air, light, and temperature) in an individually labeled Ziploc^®^ bag and stored in a container. It is recommended that samples be stored in a dark place to reduce the risk of exposure to light [81] while stored at room temperature. It is worth noting that hair samples can be stored for months to years without significant changes in hormone levels. 

Previous reports have illustrated three main methods to grind or finely cut hair into small pieces for cortisol extraction: (a) with surgical scissors [8,98,107]; (b) with a bead beater [108,109]; and (c) with a ball mill [57,109]. Each of these methods is reported to have its limitations. Notably, none of the previous documents had to address methodological concerns concerning when and how glucocorticoids were sequestered into hairs. However, there are significant distinctions between using hair analysis for glucocorticoids and for forensic purposes to determine prior stress [36]. The main differences between these methods included particle size [77], mass homogenization, time savings [110], hair nature, labor and cost efficiency, availability of necessary facilities in laboratories, and hygiene, which could potentially affect the amount of cortisol extraction [8,107,109]. Nonetheless, the study designs and laboratory procedures used were quite different, and this made it difficult to do rigorous analysis. The posterior vertex of the head was consistently sampled in human studies, but non-human studies seemed to pick locations at random or out of convenience (e.g., studies in dogs have sampled backs, shoulders, chests, and legs, depending on the research group and study) [36]. The hair follicles would have had to be ground into the smallest possible particles to achieve maximum cortisol extraction from the hair shaft. In addition, it was found that the more efficient cortisol extraction from the hair shaft resulted in better accuracy of the methodology and was more reliable [77]. In contrast to the surgical scissors’ method, the bead beater method reduced contamination and was found to be less time-consuming when multiple hair samples were processed. In addition, studies using surgical scissors reported lower overall cortisol concentrations [77,111], except for the study by Slominski et al. [112], which reported a similar amount of cortisol extracted using the milling method and finely cut hair samples. 

#### 3.3.4. Sex and Age

Sex and age are the two most important factors that influence hair cortisol/corticosterone levels. Although there is not much information in the literature about the effect of sex on hair cortisol/corticosterone levels, some studies found no differences in the levels of the hormones in different sexes in human teeth [33] and the hair of pigs [75] and cattle. [75,76]. Heimbürge et al. [76] concluded that the effect of sex on hair cortisol levels appears to be inconsistent. They suggested that diverging cortisol secretion between males and females may be due to a variety of factors such as behavioral differences, body condition, and gonadal steroid metabolism [76]. Age has appeared to have a significant effect on hair cortisol levels, as reported by Ghassemi Nejad et al. [107] and Heimbürge et al. [76]. While Ghassemi Nejad et al. [107] found higher hair and serum cortisol levels in adult cows compared to heifers, Heimbürge et al. [75] found that newborn calves had the highest hair cortisol levels compared to 6-month-old, 18-month-old, and adult cattle. In addition, Heimbürge et al. [75] revealed that piglets had the highest hair cortisol levels when compared to 10-week-old pigs, 27-week-old pigs, and sows. Furthermore, adult sows had significantly higher cortisol levels in their hair than 10-week-old pigs. They speculated that higher hair cortisol levels in younger animals could be caused by lower corticosteroid-binding globulin levels in newborn animals, resulting in increased plasma levels of free cortisol in the bloodstream [76] and thus into the hair shaft. Further research should be carried out to determine the effect of age on stress hormones in various animal species.

#### 3.3.5. Extraction Method

Using the previous methodology introduced by Davenport et al. [58], we developed an extraction method for measuring cortisol [8] and corticosterone [22] levels in our laboratory. Instead of providing a lengthy protocol, we have provided the infographic below as an example to help better understand the methodology.


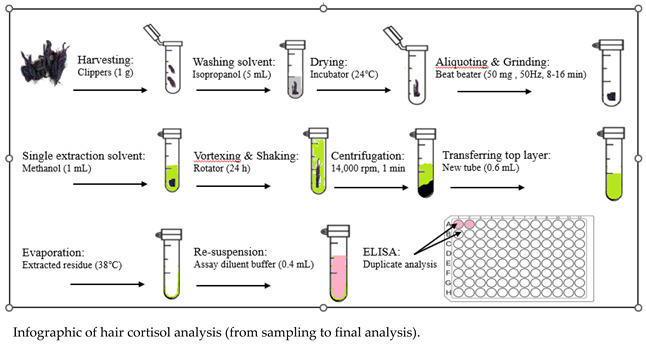


## 4. Assay Methods for Detection of Stress Hormone

There are several methods, such as immunoassay, high-pressure liquid chromatography (HPLC), mass spectrometry (MS), and fluorescence detection, that have been used to determine glucocorticoid concentration in several types of biomatrices (liquids and non-liquids). However, the immunological assay is one of the common methods used and its methodology is based on binding between an analyte and an antibody [9]. There are different forms of immunoassay, such as enzyme-linked immunosorbent assay (ELISA), radioimmunoassay (RIA), enzyme immunoassays (EIA), and multiplex immunoassay. We explain and compare the most reliable assay methods here, although other methods such as gas chromatography (GC) and HPLC can also be used [9].

### 4.1. Mass Spectrometry

The MS methods are recognized as highly specific and sensitive methods among laboratory assays. One model of gas chromatography/mass spectrometry (GC/MS) is mostly used to detect many types of metabolites and chemicals in hair with a detection limit of about 0.03 ng/mg [80,113], and another model (HPLC/MS or LC /MS), which is used to measure cortisol in the hair matrix [114,115].

### 4.2. Radioimmunoassay

The RIA is a highly sensitive test method for measuring the concentration of substances. According to the description of Accorsi et al. [98], the RIA assay has a sensitivity of 0.26 pg/mg and a specificity of 100% for cortisol. In the RIA assay, a radioisotope is attached to an antigen of interest and bound to its complementary antibody. A sample containing the antigen of interest is then added. It competes with the radioactive antigen. After unbound antigens are washed away, the radioactivity of the sample is measured. The amount of radioactive signal is inversely related to the amount of target antigen. Working with this assay requires special precautions and experienced technicians [113] because radioactive substances (health risks) are used in this method [82,114]. Although the sensitivity of the RIA assay is high and it is suitable for the detection of cortisol, its use is increasingly restricted by some government regulations to choose a more user-friendly and safer assay [9,47], so the RIA is an old assay and rarely used due to the risk of radioactive substances. The characteristics of the three RIA, EIA, and Luminex methods are summarized in Table 3 [116,117].

### 4.3. Enzyme-Linked Immunosorbent Assay or Enzyme Immunoassays

The ELISA and the EIA methods are based on enzyme immunoassays and the terms are interchangeable, but most often EIA refers to a competitive ELISA and the term ELISA refers to a sandwich ELISA assay. A sandwich ELISA refers to the antibody binding to two sites on the antigen and is more sensitive, but a competitive ELISA is less prone to experimental error because it requires only one binding site on the antigen. It is faster, more flexible, and has good reproducibility [116]. The ELISA or EIA assay is widely used among researchers because it has an acceptably high sensitivity, provides the result in a short time, and is inexpensive compared to the other aforementioned techniques. The details of the ELISA method were first explained by Engvall et al. [118]. The method allows the analysis of biomatrices immobilized in microplate wells using specific antibodies. Among the various formats of ELISA methods available, the sandwich ELISA assay, indirectly immobilizes and indirectly detects the target antigen.

### 4.4. Multiplex Immunoassays

A multiplex assay is another type of immunoassay that can measure multi-biomarkers at the same time in a single experiment [119]. It is introduced in a different format based on the utilization of flow cytometry, chemiluminescence, or electrochemiluminescence technology [120]. Multiplex arrays have several advantages over conventional ELISA, including (a) high-throughput multiplex analysis; (b) lower sample volume requirements; (c) time and cost efficiency; (d) the ability to determine the concentrations of an inflammatory molecule along with other molecules; (e) the ability to perform repeated measurements of the same cytokine panels in the same subjects under the same experimental assay conditions; (f) the ability to detect different proteins with a wide dynamic concentration range [120]. Disadvantages of multiplex assays include (a) the need for specialized equipment compared to ELISA kits [121]; (b) LUMINEX assays use fluorescence as a reporter system, whereas ELISAs use enzymatic amplification of a colorimetric substrate [121]. Performing multiplex assay requires experience and expertise.

The ELISA assay is designed to detect a single analyte (e.g., cortisol) in a single biomatrix (e.g., hair) and for analyzing in parallel, needs several experiments, which increases the cost of experiments and also increases data errors among experiments [9]. Alternately, it is very complicated to understand how different analytes (e.g., multi-biomarkers such as cortisol, cortisone, estrogen) interact with each other in a single biomatrix sample via traditional singleplex ELISA assay. However, multiplex assay allows for the measurement of several analytes in a single sample at once and the result is more biologically informative [9].

We recommend that future studies focus on the newly discovered stress indicators in animals, such as the expression of the heat shock protein gene in peripheral blood mononuclear cells and hairs [122,123] and a discussion of measurement techniques.

## 5. Conclusions

We outline the obtained conclusions of this review as follows:

First and foremost, we outlined the stress definition and classification, concluding that regardless of stress source (i.e., physiological, psychological, environmental, etc.) and type of stress (acute and chronic), a similar scenario is elevating stress hormones, specifically cortisol and corticosterone, resulting in the same pattern of elevated in body matrices and body endpoints (e.g., saliva, milk, hair, urine, feces, sweat, fins, etc.). Second, we discussed the pros and cons of each biological matrix in different animal species to be used as a possible biomarker of stress, with an emphasis on hair. Given this information, we concluded that depending on the type of acute or chronic stress and the purpose of the research, we can use the available matrix for each animal species. Considering that different animal species might provide different matrices for measuring chronic stress, it is important to find new alternatives to the pool of existing matrices. Third, we have discussed the method of sample collection, the body regions to be selected, color, sex and age effects, and the method of analysis of cortisol/corticosterone in different body indices with a focus on hair in various animal species that may be used from time to time. Given this, we outlined the effects of the aforementioned factors on hair cortisol levels, concluding that some of them are influential factors in influencing hormone levels in the hair. Regardless of the technique used for fixed indices such as hair, wool, feathers, etc., the contribution of sebum and sweat to hair cortisol levels in different species is whether sebum and sweat make a greater or lesser contribution to hair cortisol levels. Although the use of hair, wool, and feathers has shown promise for assessing prolonged stress conditions, the use of newly introduced alternatives such as fins, scales, jawbone, and earwax needs to be validated in future research. Finally, we provided information on the cortisol analysis protocol that can be used alternatively depending on the cost-effectiveness, ease of use, laboratory facilities, and skilled researchers. Overall, it is advisable to maintain the same procedure and technique for hormone determination after validation in a laboratory to avoid variations in data output and to obtain a reliable result with the lowest possible standard errors to obtain comparable results. 

## Figures and Tables

**Table 1:** Properties of hair matrix in comparison to other biological matrices where cortisol/corticosterone can be analyzed [8,37,39].

Properties			Biomatrices							
	Hair/wool	Feather	Nail/ Teeth/Scale ^1^	Earwax	Feces	Urine	Sweat	Saliva	Milk	Blood
Stressful-sampling procedure	Low ^1^	Low	No	Low	Low	Low	Low	Low ^2^	Low	High
Sampling effects on results	No	Low	No	No	No	No	Yes	Yes	No	Yes
Painful-sampling procedure	Low	Low	No	Low	Low	Low	Low	Low	Low	High
Liability to external contamination	High	High	Low	Low	High	High	High	High	Low	Low
Application acute stress	No	No	No	Yes	Yes	Yes	Yes	Yes	Yes	Yes
Application chronic stress	Yes	Yes	Yes	Yes	Yes	Yes	No ^3^	No ^4^	No ^5^	No ^6^
Possibility of repeated sampling	No	No	No	Yes	Yes	Yes	No	Yes	Yes	Yes
Liability to blood contamination of samples	Yes	No	No	No	Yes	Yes	Yes	Yes	Yes	No
Effect of pH on composition	No	No	No	Yes	Yes	Yes	Yes	Yes	Yes	Yes
Affected by location of sample collection	Yes	No	No	No	Yes	Yes	Yes	No	No	No
Need for vet personnel for sample collection	No	No	No	Yes	No	Yes	No	No	No	Yes
Time periods of cortisol represented	Weeks, Months, Years	Weeks, Months, Years	Weeks, Months, Years	Weeks, Months	Two to four days	One to two days	Single point, hour	Single point, hour	4–10 h	Single point, hour
Measuring cortisol forms	Unbound	Unbound	Unbound	Unbound	Unbound	Unbound	Unbound	Unbound	Bound and unbound	Bound and unbound
Storage condition	Room temperature	Room temperature	Refrigeration, freezing	Refrigeration, freezing	Refrigeration, freezing	Refrigeration, freezing	Refrigeration, freezing	Refrigeration, freezing	Refrigeration, freezing	Refrigeration, freezing
Analytical costs	High	High	High	Medium	Medium	Medium	High	Medium	Medium	Medium

^1^ Depending on the species. ^2^ In mice and rats that may be stressful. ^3,4,5,6^ Series of measurements could provide information about chronic stress.

**Table 2:** * The summary of the capability of the hair matrix for measuring cortisol in species.

Strengths	Considerations
The procedure of hair sampling is a non-invasive method and simple.	Difficulty in the collection of short hair species or offspring.
At most 1g of hair sample is required.	Cannot access hair loss people and animals.
Ease of storage and stability at room temperature for years and hair is resistant to chemical decomposition rather than other biological matrices.	Requiring to wrap hair samples in aluminum foil to maintain the integrity and to avoid extra contamination while storing at room temperature.
Retrospective calendar of cortisol secretion - provides a window to the past.	Hair samples do not reflect the immediate or recent exposure to cortisol secretion.
Hair characteristics including hair color [7] do not influence the amount of cortisol. Sampling is possible by researcher.	Possible confounding factors: age, sex, hair growth rate, and body weight.
Easily can be transferred to the laboratory for analysis.	Still need to determine the most effective cortisol extraction method (methods of cortisol extraction differ between studies).

* Ataallahi et al. [9]; Smyth, [90].

**Table 3:** Comparison of (radio-immunoassay) RIA, (enzyme immunoassays) EIA assay and Luminex ^1^ methods of hormone detection.

Characters	RIA Assay	EIA Assay	Luminex Assay
Easy to perform with a simple procedure	No	Yes	Yes
Immunoassay type	Yes	Yes	Yes
Antigen and antibody reaction	Yes, by immune methods	Yes	Yes
High specificity and sensitivity	Yes	Yes	Yes
Useful in diagnosis and research	Yes	Yes	Yes
Generally safe and eco-friendly	No	Yes	Yes
Radioactive substances requirement	Yes	No	No
Cost-effective assay	No	Yes	Yes
Reagents cost	High cost	Low cost	Low cost
Time duration of the experimental procedure	Time consuming	Short and fast	Save time and money compared to multiple single-analyte ELISAs
An efficient and highly skilled handler is needed	Yes	No	No
Disposal requires special care	Yes, to avoid radiation exposure	No	No
Disposal of waste is simple Measures multiple analytes in one sample Track biological interactions in real time	No No No	Yes No No	Yes Yes Yes

^1^ Luminex xMap technology is a bead-based multiplexed immunoassay system in a microplate format. The system can simultaneously detect many targets in a single sample—up to 500, depending on system design.

## Data Availability

Not applicable.
